# Participation du patient Marocain atteint du cancer au choix thérapeutique: résultat d'une enquête réalisée auprès de 272 patient

**DOI:** 10.11604/pamj.2015.22.174.7040

**Published:** 2015-10-22

**Authors:** Anwar Boukir, Ilham Azghari, Mustapha El Kabous, Khalid Jouid, Saber Boutayeb, Ibrahim El Ghissassi, Hind Mrabti, Hassan Errrihani

**Affiliations:** 1 Service d'Oncologie Médicale, Institut National d'Oncologie, Centre Hospitalier Universitaire Ibn Sina, Faculté de Médecine et de Pharmacie, Université Mohammed V, Rabat, Maroc; 2 Service d'Hépato-gastro-entérologie, Centre Hospitalier Universitaire Ibn Sina, Faculté de Médecine et de Pharmacie, Université Mohammed V, Rabat, Maroc; 3 Service de Dermatologie, Centre Hospitalier Universitaire Ibn Sina, Faculté de Médecine et de Pharmacie, Université Mohammed V, Rabat, Maroc

**Keywords:** Shared décision making, Maroc, cancer, Shared décision making, Morocco, cancer

## Abstract

**Introduction:**

La décision médicale partagée ‘Shared decision making’ est un concept qui se développe depuis les années 1990. Il donne aux patients le soutien nécessaire pour exprimer leurs préférences et partager la décision médicale. Cette étude cherche à estimer le degré de participation du patient Marocain atteint de cancer au choix thérapeutique.

**Méthodes:**

Cette enquête a été réalisée auprès de 272 malades sous chimiothérapie pour une pathologie cancéreuse sous forme d'un entretien verbal basé sur un questionnaire. Les patients ont été sélectionnés selon un mode d’échantillonnage aléatoire, le nombre de patients a été choisi pour une marge d'erreur de 5% et un seuil de probabilité qui approxime les 90%.

**Résultats:**

Seulement 5.5% des patients dans l'enquête ont participé activement dans le choix thérapeutique. Pour 94% des patients de l’échantillon la stratégie thérapeutique adoptée par le médecin est la bonne et représente l'option optimale. Les principales causes retrouvées qui expliquent la non participation à la décision thérapeutique sont le bas niveau d'instruction, la non réceptivité à l'information ainsi que des défauts majeures dans la transmission et la perception de l'information.

**Conclusion:**

Ces résultats prouvent que la relation médecin malade dans notre contexte baigne toujours dans le modèle paternaliste. La responsabilité de la décision thérapeutique est le plus souvent laissée au médecin. Il est nécessaire d'informer et d'impliquer le patient de façon active dans le choix thérapeutique afin de mieux sauvegarder la relation médecin-malade qui doit être fondée sur la confiance ainsi que sur une approche participative.

## Introduction

La notion de participation des patients dans la décision thérapeutique n'est pas bien comprise. Elle est souvent assimilée au respect du traitement médical et des ordres du médecin, et moins souvent vue comme un dialogue interactif ou le patient peut donner son avis [1]. La décision médicale partagée ‘Shared decision making’ est un concept qui se développe depuis les années 1990. Ce dernier va au delà du partage de l'information entre praticiens et patients et donne aux patients le soutien nécessaire pour exprimer leurs préférences et partager non seulement la décision médicale mais tout le processus qui conduit vers cette dernière [2]. Le but de cet article est de présenter les résultats de notre enquête sur la participation des patients cancéreux marocains au choix thérapeutique.

## Méthodes

Il s'agit d'une enquête réalisée dans le service d'Oncologie Médicale à l'Institut National d'Oncologie à Rabat au Maroc, sous forme d'un entretien verbal basé sur un questionnaire. 272 patients ont été sélectionnés selon un mode d’échantillonnage aléatoire pour une marge d'erreur de 5% et un seuil de probabilité qui approxime les 90%. Les critères d'inclusion étaient: un âge supérieur à 15 ans, un état neurologique et psychique permettant un transfert fluide d'informations et l'obtention du consentement éclairé du patient. Cette étude a concerné des patients suivis pour une pathologie cancéreuse sous chimiothérapie. Les questions portaient sur le profil sociodémographique des patients, le degré de leur participation au choix thérapeutique et ses formes, et leur propre jugement par rapport à celle-ci. L’évaluation objective de la participation des patients au choix thérapeutique étant difficile à mesurer, elle s'est fiée aux réponses des patients aux questions directes reflétant l'impression qu'ont ces derniers par rapport à leur propre implication dans le processus thérapeutique. Le questionnaire a été conçu par l’équipe médicale du service d'Oncologie Médicale devant la constatation du manque d'implication des patients dans le processus de choix thérapeutique, dans l'optique d'une meilleure appréciation de ce fait. Il a été validé par le comité d’éthique de l'Institut. Conçu en langue française, le questionnaire a été administré par le médecin traitant en fonction de la langue du patient (arabe, dialecte marocain, amazigh…).

## Résultats

**Figure 1:**
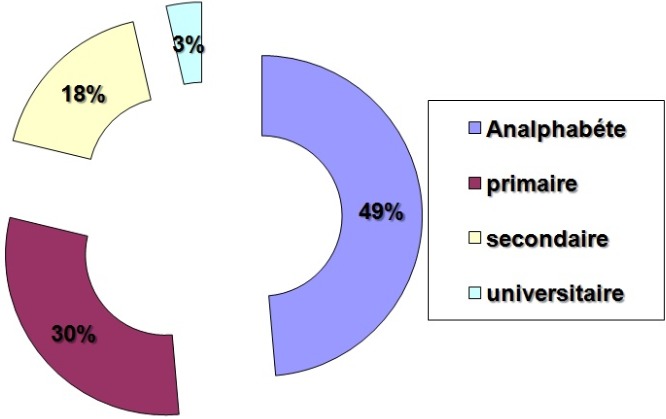
Le niveau d'instruction chez la population des patients interrogés

L’échantillon comportait 64% de femmes et 34% d'hommes. L’âge moyen des patients était de 50 ans et 72% d'entre eux avaient moins de 60 ans. 66% des patients provenaient d'un milieu urbain alors que 34% des patients habitaient le milieu rural. En ce qui concerne le niveau d'instruction, 79% des patients étaient analphabètes ou avaient un niveau d’études primaires. Seuls 18% des patients avaient un niveau secondaire et 3% un niveau universitaire (Figure 1). 82% des consultations se sont faites en la présence d'accompagnants. Dans 23% des cas, c'est l'accompagnant qui était le principal interlocuteur et le preneur de décision. A la question «avez-vous participé au choix thérapeutique», seulement 5,5% (n = 15) des patients ont exprimé avoir participé activement. Une participation active a été retenue lorsque le patient: dit être au courant des options thérapeutiques disponibles; est capable de défendre le choix thérapeutique actuel; exprime des préférences si plusieurs options sont présentées. Un pourcentage important de patients considère le consentement comme une forme de participation à la décision thérapeutique, mais l'adhérence passive à la décision a été considérée comme une non participation. Les 15 patients ayant participé activement à la décision thérapeutique se sont renseigné sur les options disponibles, sur le risque-bénéfice et les effets indésirables, l'altération de la qualité de vie, le pronostic et le coût, avant d'exprimer leur préférence en faveur d'une option thérapeutique.

Nous notons que chez deux patientes suivies pour un cancer du sein le choix thérapeutique a consisté en la décision de réaliser un traitement conservateur. Un patient suivi pour un sarcome a refusé l'amputation en assumant les risques. Trois patients ont choisi le rythme de l'administration de la chimiothérapie et deux patients ont préconisé la voie per os. Un patient en situation métastatique, a refusé, après information, l'administration d'une nouvelle ligne de chimiothérapie. Deux patients dans la même situation ont choisi de subir d'autres lignes de chimiothérapie. Malgré la non participation au choix thérapeutique de la majorité des patients interrogés, 94% des patients ont déclaré faire confiance à leurs praticiens et considèrent que la décision prise représente la solution thérapeutique optimale. D'un point de vue médical, la décision thérapeutique a respecté les recommandations des sociétés savantes dans 98% des cas. Ce résultat prouve que l'absence de participation du patient à la décision thérapeutique n'affecte pas l'exactitude et l'efficacité de cette dernière. Cette absence de participation semble arranger le déroulement de la prise de décision en la libérant des contraintes en rapport avec les exigences des patients.

## Discussion

Dans ce chapitre nous allons essayer d'expliquer ces résultats. Le premier paramètre entrant dans l'analyse des résultats de notre enquête est le profil socio-démographique des patients. Il n'y avait pas de différence significative des résultats de l'enquête en fonction de l’âge, du sexe ou du milieu de provenance. Cependant, un plus haut niveau d'instruction était lié à une meilleure participation au choix thérapeutique. En effet, 11 patients parmi ceux qui ont participé activement à la décision thérapeutique appartiennent au 21% des patients présentant le meilleur niveau d'instruction. Le déroulement de la consultation lors de laquelle sont exposées les options thérapeutiques est également un élément clé dans la participation du patient au choix. Dans ce sens, on peut dire que la présence d'un accompagnant, qui se transformait en interlocuteur principal dans 23% des cas, constituait une gêne des transferts médecin-malade. L'accompagnant peut jouer un rôle de filtre qui déforme l'information dans un sens comme dans l'autre.

**Figure 2:**
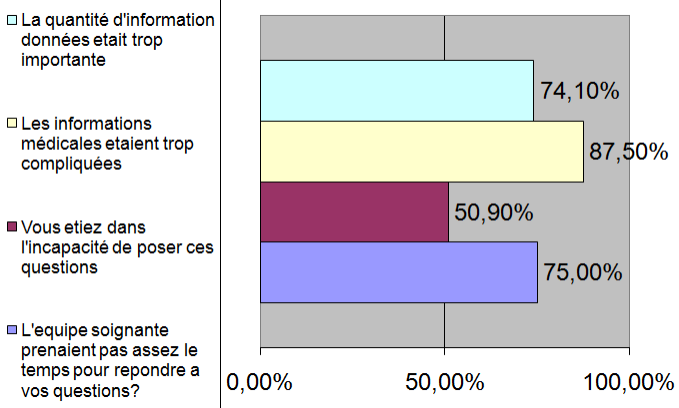
Les principales causes de la mauvaise transmission de l'information selon les patients interrogés

Le patient pris isolément, quant à lui, peut manquer de réceptivité par rapport à l'information émise. En effet, une étude menée parallèlement au même centre hospitalier, avait montré que les patients Marocains sont peu réceptifs: 39% d'entre eux n'expriment aucun désir pour recevoir des informations [3]. Le patient pointe également du doigt la non disponibilité de l’équipe soignante qui découle sur un manque quantitatif et qualitatif de transmission de l'information (Figure 2). Ce défaut de transmission de l'information prive le patient des données nécessaires pour participer activement à la décision thérapeutique. Parfois, et malgré un échange réel d'information, la perception de cette dernière par le patient ne reflète pas tout à fait la réalité. Une enquête réalisée sur le même échantillon démontre que le pronostic médical est mal assimilé dans 86% des cas, il existe une surestimation du pronostic dans 18% des cas contre 68% des cas ou existe une sous estimation de ce dernier [3]. Une mauvaise assimilation du pronostic va certainement biaiser la participation à la décision thérapeutique. Un patient qui ne maîtrise pas les enjeux d'une telle décision sur sa santé ne pourra pas être considéré comme un participant actif.

## Conclusion

La relation médecin malade dans notre contexte baigne toujours dans le modèle paternaliste. Seulement 5,5% des patients ont participé activement à la décision thérapeutique les concernant et 94% des patients expriment leur confiance totale en la décision prise par leurs médecins traitants. Les principales causes retrouvées et qui expliquent cette non participation sont le bas niveau d'instruction, la non réceptivité à l'information ainsi que des défauts majeures dans la transmission et la perception de l'information. Bien qu'aucune preuve ne démontre que la décision médicale partagée ait un impact sur l’état de santé du patient, l'application de ce principe consolide la dimension humaine dans la prise en charge du malade et veille à l'application d'une médecine fondée sur les preuves et assure un meilleur engagement des deux protagonistes à savoir le médecin et le malade.
